# Child and adolescent mental health services in Aotearoa New Zealand

**DOI:** 10.1192/bji.2025.5

**Published:** 2025-05

**Authors:** Edward Miller, Anthony James

**Affiliations:** 1 Honorary Lecturer, Division of Psychological Medicine, University of Auckland, Auckland, New Zealand. Email: emil673@aucklanduni.ac.nz; 2 Honorary Senior Lecturer, Highfield Unit, Warneford Hospital, Oxford, UK

**Keywords:** Transcultural psychiatry, child and adolescent psychiatry, mental health services, New Zealand, global mental health

## Abstract

This paper aims to give an overview of child and adolescent mental health services (CAMHS) in Aotearoa New Zealand. We provide a brief overview of the demographics of the country and include the most up-to-date epidemiological data on child and adolescent mental health. To meet these psychiatric needs, we consider the present workforce, governance, funding and structure of CAMHS. Current psychiatric practice is heavily influenced by the country's unique history and cultural mix. Aotearoa New Zealand is noted for outstanding epidemiological research.

Aotearoa New Zealand is a small country comprising two main islands located in the southwestern Pacific Ocean. The indigenous people of the land are known as the Māori and their language is called Te Reo, which means ‘The Language’. Aotearoa is the Te Reo name for New Zealand, which translates as ‘land of the long white cloud’. Aotearoa New Zealand was colonised by Europeans from the 18th century onwards and today has a multicultural population of about 5 million people, of which one-quarter are under 19 years old. This quarter comprises New Zealanders of European origin (46%), Māori (27%), Pasifika (a Polynesian language term used as an umbrella definition of people of Pacific Island descent, including those born in Aotearoa New Zealand but not Māori) (10%) and people of Asian ethnicity (17%). English, Te Reo and New Zealand Sign Language are the official languages of the country.

## Epidemiology

The demand for CAMHS services in Aotearoa New Zealand is high, with increased complexity and demand after the COVID-19 pandemic, particularly among minority ethnic groups.^1^ Unfortunately, the country has one of the highest youth suicide rates among Organisation for Economic Co-operation and Development/European Union (OECD/EU) countries, ranking the highest in 2010 and second highest in 2020, with disproportionately higher rates among youth of Māori and Pasifika descent.^2^ Aotearoa New Zealand also ranks 35th out of the 41 OECD/EU countries for child and youth well-being outcomes such as having enough food, and these outcomes are poorest for Māori and Pasifika child and youth.^3^ Recent data indicate that 21% of young people aged 15–24 reported psychological distress in 2022–2023, compared with just 5% in 2011–2012, although funding has been cut from 13% of the mental health budget down to just 10%.^4^ Unfortunately, Aotearoa New Zealand's only national prevalence survey of mental disorders (Te Rau Hinengaro: the New Zealand Mental Health Survey) was completed in 2006 and was based on 2003–2004 data. It is now 20 years out of date and surveyed only people aged 16 years and over, meaning that there are no useful data on prevalence of mental health problems among the country's children and young people. There is strong advocacy within Aotearoa New Zealand at present for better mental health data collection for children and young people, and this was an electoral issue at the most recent national elections in 2023. A 2018 government inquiry, He Ara Oranga (‘Pathways to Wellness’), into the functioning of the whole mental health sector identified several areas of reform, including upcoming changes to the Mental Health (Compulsory Assessment and Treatment) Act 1992 and the establishment of a new Mental Health and Wellbeing Commission.

Māori and Pasifika youth are reported to have much higher rates of mental health problems in general compared with other ethnic groups.^5^ Despite this, there remain many cultural barriers to their accessing mental healthcare, with Māori access rates across all age ranges in 2021 being only 5.1%, and Pasifika and Asian rates being 2.4 and 1.4% respectively, compared with a New Zealand European rate of 5.7%.^1^ Māori are also subject to disproportionately higher use of community treatment orders (CTOs), and there are significant cultural differences in how mental illnesses are viewed. For example, some types of psychotic-like experiences occurring in Māori youth may in fact be spiritually or culturally understood.^6^ Moreover, the effective delivery of health services to Māori centres on recognising the role of including the extended family (whānau and hapu) in both the health of an individual (tangata whaiora) and in their engagement with services. This can commonly follow the Te Whare Tapa Whā model, which aims to address spiritual well-being (taha wairua), mental and emotional well-being (taha hinengaro), physical well-being (taha tinana) and family (whānau) and social well-being (taha whānau), which are all mediated by connection to the land (whenua).^7^ CAMHS in Aotearoa New Zealand particularly value practices that enhance and protect Mana – one's individual agency – which stems from social relationships, connection to the land and relationship with Atua (the Divine), through incorporating skills such as listening to and valuing the perspectives of the children and young people and whānau who access services.^1^

## Workforce, structure and governance

It has been reported that Aotearoa New Zealand should aim for a minimum of 4.0 full-time equivalent (FTE) child and adolescent psychiatrists per 100 000 general population, but currently there are only 1.0 FTE.^8^ The most recent data show that there are only 46 child and adolescent psychiatrists in the whole country, and the workforce is ageing, with the majority located in urban areas, and only a small number of child and adolescent psychiatry trainees advancing. Presently, the majority of child and adolescent psychiatrists work in the public system, with less than 10% in private practice, and there is a relative weight towards public health and social services in Aotearoa New Zealand rather than privatisation. In 2022–2023, public clinical services for children and young people were understaffed by 13%, mostly in clinical roles, with high rates of general staff turnover.^1^

The most recent data on mental health funding (from 2021–2022) indicate that 13% of overall government health funding is spent on infant and child and adolescent mental health services (NZ$228 million), with the majority (NZ$165 million) going towards funding public services and NZ$63 million funding non-governmental organisations (NGOs) and school-based programmes.^1^ Public funding is primarily divided between in-patient and community out-patient mental health services, including general and specialist services. Other publicly funded community services that are commonly accessed by young people include general practice (GP) and youth-specific integrated primary care services, which are ‘one stop shop’ type approaches.^9^

There are 20 CAMHS teams in Aotearoa New Zealand in total, which are multidisciplinary in nature and include social workers, psychologists, psychiatrists, child psychotherapists, occupational therapists and nurses. These teams deliver mental health assessment and intervention to young people and their families, but have low access rates of less than 5%.^1^ The community CAMHS teams primarily utilise the Choice and Partnership Approach (CAPA), which was originally developed in the UK and focuses on demand management and offering sustainable treatment choices for young people and their families. Other interventions offered by clinicians include parenting programmes such as Incredible Years, and therapies such as cognitive–behavioural therapy (CBT) and whanaungatanga-informed approaches, which involve helping Māori children and young people to foster and acknowledge a kinship with their ancestry and cultural land.^1^

There are presently three in-patient child and adolescent psychiatry units in Aotearoa New Zealand, which are used for acute care of severe mental illness and are generally not for lengthy stays.^1^ One is based in Auckland, with 3 FTE psychiatrists for 18 beds, including a 3-bed mother and baby unit, all of which are for supra-regional coverage across most of the North Island; one is based in Wellington, with 13 beds, which services the regional Wellington area on the North Island; and one in Christchurch, with 16 beds, servicing the entirety of the South Island. There is also a single 10-bed national secure youth forensic in-patient unit based in Wellington, which is for young people 13–17 years of age who have severe mental health or addiction problems and are currently involved with the youth justice system. These in-patient services are externally regulated for quality of care, and to ensure that children's rights are maintained, by the independent children's and young person's commission Mana Mokopuna, which is run by a board of five commissioners as an independent Crown entity.

Child and adolescent psychiatry training falls under the remit of the Royal Australian and New Zealand College of Psychiatrists (RANZCP) and its Faculty of Child and Adolescent Psychiatry. The RANZCP is a binational college committed to the training, education and representation of psychiatrists across both countries. It was founded in 1946, and the Faculty of Child and Adolescent Psychiatry had its beginnings in 1964 as the first subspecialty group of the RANZCP. Advanced training in child and adolescent psychiatry is an additional 2 years following a generalist 3-year initial programme, and trainees undertaking it work with children and young people only during their clinical rotations, alongside completing the relevant assessments and psychotherapy cases as part of this training.^10^

## Academic child psychiatry

Like its status overseas, academic child psychiatry in Aotearoa New Zealand faces a difficult plight. Aotearoa New Zealand has four academic departments of psychiatry in total, but there are limited to no opportunities for child and adolescent psychiatry trainees to participate meaningfully in relevant research, partly because of a lack of predefined pathways. There are also extremely few academic child psychiatrists in Aotearoa New Zealand, partly owing to a limitation in funding pathways.

Despite this, Aotearoa New Zealand has world-leading research in the field of child development, with the Dunedin Multidisciplinary Health and Development Study (commonly known as the Dunedin Study) and the Growing Up in New Zealand study as two examples of world-class longitudinal cohort studies. There is also a strong focus on research in the field of e-mental health, including the use of applications (apps) as primary prevention for low acuity presentations and patients on waiting lists. These include the Sparx e-therapy game for children and young people with depression (www.sparx.org.nz/) and the Headstrong mobile app for well-being (www.headstrong.org.nz/), which were both made possible through government funding and the first of which has an international trial pending.^11^

## Conclusion

Child and adolescent psychiatry in Aotearoa New Zealand is an established field but faces many challenges across its training, workforce, governance, service provision and funding. Despite this, a focus on indigenous and traditional perspectives, particularly from Māori and Pasifika peoples, and impactful areas of research are distinct areas of strength.

## Data Availability

Data availability is not applicable to this article as no new data were created or analysed in this study.
